# Machine Learning-Based Shelf Life Estimator for Dates Using a Multichannel Gas Sensor: Enhancing Food Security

**DOI:** 10.3390/s25134063

**Published:** 2025-06-29

**Authors:** Asrar U. Haque, Mohammad Akeef Al Haque, Abdulrahman Alabduladheem, Abubakr Al Mulla, Nasser Almulhim, Ramasamy Srinivasagan

**Affiliations:** 1 Department of Computer Science, College of Computer Science and Information Technology CCSIT, King Faisal University, Al AHSA 31982, Saudi Arabia; 2 Department of Networks and Operating Systems, Deanship of E-Learning and Information Technology DEIT, King Faisal University, Al AHSA 31982, Saudi Arabia; malhaque@kfu.edu.sa; 3 Department of Computer Engineering, CCSIT, King Faisal University, Al AHSA 31982, Saudi Arabia

**Keywords:** shelf life, IoT, dates, multichannel gas sensor, DHT11 sensor, gas concentrations, food security, edge impulse, machine learning (ML), relative humidity (RH), cold storage room (CSR)

## Abstract

**Highlights:**

**What are the main findings?**

- A low-cost IoT system combining multichannel gas sensors and a TinyML model was developed for the real-time shelf life prediction of dates.
- The deployed model achieved 91.9% classification accuracy and an AUC of 0.98 using data collected under cold storage and ambient conditions.

**What are the implications of the main findings?**

- Unlike traditional visual inspection methods, this system offers a scalable and objective solution for early spoilage detection.
- This system contributes to improved cold storage decision-making, reducing postharvest losses and enhancing food supply chain resilience.

**Abstract:**

It is a well-known fact that proper nutrition is essential for human beings to live healthy lives. For thousands of years, it has been considered that dates are one of the best nutrient providers. To have better-quality dates and to enhance the shelf life of dates, it is vital to preserve dates in optimal conditions that contribute to food security. Hence, it is crucial to know the shelf life of different types of dates. In current practice, shelf life assessment is typically based on manual visual inspection, which is subjective, error-prone, and requires considerable expertise, making it difficult to scale across large storage facilities. Traditional cold storage systems, whilst being capable of monitoring temperature and humidity, lack the intelligence to detect spoilage or predict shelf life in real-time. In this study, we present a novel IoT-based shelf life estimation system that integrates multichannel gas sensors and a lightweight machine learning model deployed on an edge device. Unlike prior approaches, our system captures the real-time emissions of spoilage-related gases (methane, nitrogen dioxide, and carbon monoxide) along with environmental data to classify the freshness of date fruits. The model achieved a classification accuracy of 91.9% and an AUC of 0.98 and was successfully deployed on an Arduino Nano 33 BLE Sense board. This solution offers a low-cost, scalable, and objective method for real-time shelf life prediction. This significantly improves reliability and reduces postharvest losses in the date supply chain.

## 1. Introduction

There is no doubt that adequate nutrition is essential for human beings to live a healthy life. It is considered that dates are one of the best nutrients for survival. Dates are the main food supplements consumed day-to-day amongst a large population across the globe. The date fruit is cultivated on 1.25 million hectares globally, yielding an annual production of 9.61 metric tons (MT). Saudi Arabia is considered to be one of the highest producers and exporters of dates. Saudi Arabia cultivates in an area equal to 0.15 million hectares, producing 1.54 MT [1]. Date palms are seasonal trees, and, hence, preserving the harvested product is much needed to maintain supply throughout the year, enhancing food security. Most of the date fruits’ palm cultivars are consumed at the fully ripened stage, known as the Tamr stage. Dates are subjected to post-harvest losses due to factors which include the following: erratic environmental conditions, insect infestation, and microbial activity.

Dates, like other stored fruit products, are usually accompanied by respiration, evaporation, and physiological changes due to moisture losses, which could induce heavy losses reaching up to 40%. Therefore, suitable storage facilities are essential for extending the shelf life of date fruits [2]. In order to make customers satisfied with the product, it is absolutely crucial to maintain freshness throughout the supply chain. Usually, cold storage provides the ambient conditions necessary to preserve the harvested dates. Relative humidity (RH), and temperature are the core parameters for sustaining the nutrient contents. Modern cold storage is used in date fruit storage, and precise control of the RH is empirically used at a small scale or at laboratory level.

Several studies have explored the impact of environmental control in cold storage on the shelf life of dates and other perishable products. These efforts primarily focus on regulating temperature and humidity to slow physiological degradation, but they often do not adapt dynamically to the actual conditions or remaining shelf life of the stored dates. Fruits are typically stored at non-freezing temperatures to preserve tissue integrity and prevent cell structure disintegration and postharvest damage [3]. Date palm fruits harvested at the Tamer stage are generally kept in storage conditions that are necessary to prolong their shelf life [4]. Key challenges during storage include fruit weight loss and microbial degradation, such as bacterial fermentation and postharvest fungal infections [5]. Low-temperature storage also helps preserve the non-esthetic quality traits of fruits, including texture, nutritional value, aroma, and flavor [6].

In the storage of fresh fruits, temperature is closely correlated with respiration rates. These rates can be reduced by lowering the temperature to an optimal threshold, depending on the specific produce. Lower respiration rates slow the kinetics of biochemical reactions, including those affecting product quality [7]. Temperature thus plays a critical role in determining the shelf life of food products by directly influencing these biochemical activities [8]. Through respiration and microbial degradation, nutrients are broken down into simpler compounds, often leading to reductions in both the quantity and quality of the stored food [9]. In the case of date fruits, weight loss and microbial spoilage—such as yeast and bacterial fermentation, as well as postharvest fungal infections—are major limiting factors for prolonged storage [5,10]. Low-temperature environments also help preserve non-esthetic quality attributes, such as texture, nutritional value, aroma, and flavor [6,11]. Therefore, dates must be kept at low temperatures immediately after harvesting to inhibit microbial growth and insect activity. Additionally, cold storage reduces the vapor pressure differential between the atmosphere and the fruit surface, effectively minimizing moisture loss [12].

Real-time environmental monitoring within cold storage facilities has become feasible through the Internet of Things (IoT) [13,14,15,16,17,18]. However, most existing IoT-based systems focus solely on environmental data collection and do not provide direct shelf life estimation. For example, the IoT-based monitoring framework proposed in [19] uses low-cost gas sensors for food quality evaluation, yet it cannot simultaneously detect multiple volatile gases released during spoilage. This limitation reduces its applicability in complex environments where multiple gas interactions are indicative of food quality deterioration.

When food is stored at refrigerator temperatures of 5 °C for a year, it is less likely to become spoiled, and the aerobic bacteria, yeast, and fungi are less active [20]. In contrast, low-temperature storage necessitates high humidity in order to prevent water loss and preserve the fruit’s characteristics. An RH of 90% is typically the most suitable for fruit storage because the majority of fungi stop growing under conditions of less than about 90% RH, and some fungi can grow at 85% RH [6,21]. Product quality suffers because of storage-specific variations in humidity and temperature in the CSR [19,22]. Unwrapped agricultural products like fruits, vegetables, and other foods are prone to lose moisture, which also reduces the product’s shelf life through dehydration and deterioration. As a result, humidification systems might be able to help cut down on these losses and reduce bacterial growth [14,23]. Controlling a high RH, ideally between 90% and 95%, could prevent water loss during the postharvest cold storage of fruits [5]. The microbial quality of agricultural products like fruits and vegetables did not change when they were humidified; this might be because drinking water contains ozone [24,25,26,27]. In most cases, food humidification systems in CSRs increase the amount of water in the air to reduce the difference between the air and the food surface’s water vapor pressures, helping to retain quality [28,29,30].

From the above discussion, it is evident that maintaining optimal temperature and RH is vital for preserving date palm fruit quality and extending its postharvest shelf life [31,32]. Accurate shelf life estimation is crucial, as it can guide the regulation of temperature and humidity within CSRs to align with the actual condition of stored dates. However, existing methods for shelf life assessment in cold storage environments are either destructive, expensive, or require invasive interventions that disrupt CSR operations. Therefore, any scalable shelf life estimation technique should be non-invasive, cost-effective, and compatible with existing storage infrastructure.

Two notable approaches exemplify the current technological direction. Mohammed et al. [33] used artificial neural networks to predict date quality based on electrical properties, achieving high accuracy but relying on frequent, contact-based LCR measurements that are unsuitable for real-time use. In another approach, Mohammed et al. [34] utilized multispectral sensors with TinyML to estimate shelf life under modified atmosphere packaging. While it is compact and non-destructive, this method depends on custom packaging and specialized hardware, limiting its deployment in standard CSRs.

To overcome these limitations, our work proposes a machine learning-based IoT architecture employing low-cost multichannel gas sensors and measuring spoilage-indicating gases such as CH_4_, NO_2_, and CO, along with temperature and RH. The system enables real-time, non-invasive shelf life estimation using edge-deployable ML models without altering the CSR environment or packaging.

We hypothesize that this combination of low-cost gas sensors and lightweight ML models can accurately classify the shelf life stage of stored dates, making it suitable for real-world deployment in cold storage facilities as well as in ambient conditions. The proposed solution offers a scalable, smart, and automated method to support supply chain efficiency and reduce postharvest food loss contributing to improved food security.

The rest of the paper is organized as follows: Section 2 outlines the materials used and methods adopted in this research. Section 3 discusses results and its significance. Section 4 presents the conclusion and suggests future research directions

## 2. Materials and Methods

**Figure 1:**
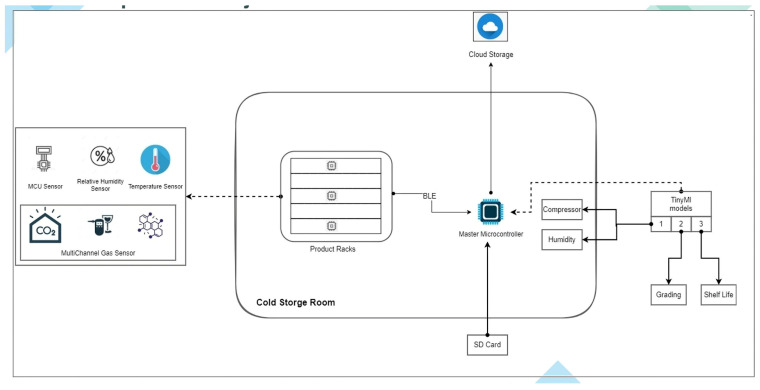
Architecture of the proposed shelf life estimation system, illustrating the major hardware components and their interactions.

Figure 1 shows the architecture of the overall system setup in the CSR of the Date Research Center of King Faisal University. The system contains a multichannel gas sensor to detect gas emissions. There is a humidity sensor to measure moisture levels along with a temperature sensor to track the temperature variations. These sensors collect real-time data from the CSR, where products are stored, and send it to a master microcontroller via Bluetooth Low Energy (BLE). The dates used in this experiment were harvested at the fully ripened Tamr stage and placed into CSRs. Data collection began shortly after, following setup and sample arrangement. The machine learning model was applied from the start of sensor data acquisition and remained active throughout the postharvest monitoring period, which extended over several months.

The following hardware and software components have been used:

(i) The Multichannel Gas Sensor V2: This sensor incorporates four elements, namely GM102, GM302, GM502, and GM702. This low-cost sensor was used in the experiment because each element is specifically designed to detect a particular gas or group of gases. For instance, GM102 is designed to detect hydrogen (H_2_), GM302 can detect carbon monoxide (CO) and nitrogen dioxide (NO_2_) gases, GM502 can sense ammonia (NH_3_) and hydrogen sulfide (H_2_S), while GM702 is sensitive to methane (CH_4_) gas.
(ii) The DHT11 sensor: This was used to measure temperature and humidity. It uses a capacitive humidity sensor and a thermistor to measure the surrounding air, and emits a digital signal on the data pin (no analog input pins needed).
(iii) Arduino boards: This was used to design the IoT-based estimator of a variety of controllers and microprocessors. Sets of digital and analog input/output (I/O) pins can be used to connect the boards to various expansion boards and other circuits. The boards have serial communications interfaces, some of which are USB (Universal Serial Bus), and they can also be used to load programs.
(iv) Standard API: Using a standard API that is also referred to as the Arduino language and is based on the processing language and utilized with a modified version of the Processing IDE, the microcontrollers can be programmed using the C and C++ programming languages.
(v) TinyML kit: This contains Arduino Nano 33 BLE Sense, which integrates a combination of six or seven sensors in one shield. The Arduino Nano 33 BLE Sense combines a small form factor, a wide range of environmental sensors, and the ability to run AI with TinyML and TensorFlow Lite.

**Figure 2:**
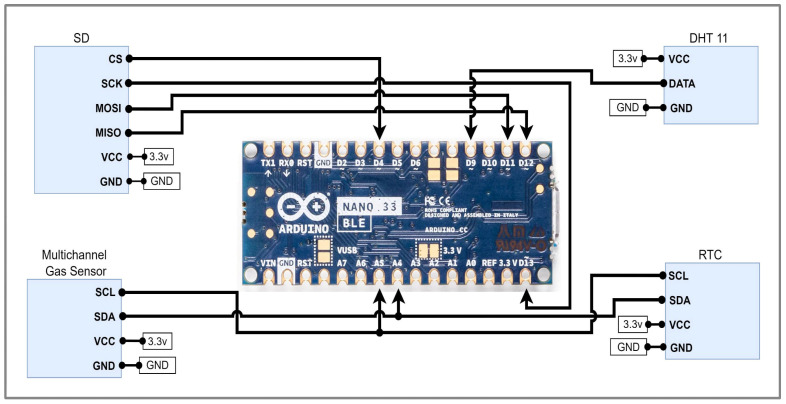
Circuit connections between Arduino and sensors.

The circuit diagram in Figure 2 illustrates a sensor-based setup built around the Arduino Nano 33 BLE microcontroller. Multiple sensors and peripherals are integrated into the system to gather and show environmental data. A multichannel gas sensor is linked to the microcontroller to detect various gases, with power supplied at 5 V and data communicated via SPI. A DHT11 sensor is wired to provide real-time humidity readings, using a DATA pin along with VCC and GND connections, as shown in Figure 2. Along with that, the circuit becomes attached to a Real-Time Clock (RTC) module for accurate timestamping of the collected data, communicating over the I2C protocol via SCL and SDA lines. Moreover, an OLED display is connected through the I2C (SCL and SDA) protocol to display the estimated shelf life of the harvested items. Last but not least, an SD card was wired through the SPI interface to store the collected data for analysis. This setup allows for the real-time monitoring of environmental conditions, with data logging capabilities and a visual display for immediate feedback, making it ideal for applications in climate control, air quality monitoring, and smart storage systems. Table 1 shows various modules and related information.

The IoT-based system was set up at the Date Palm Research Center (DPRC) at King Faisal University, Al Ahsa, Saudi Arabia, to collect valuable research data. The DPRC houses the CSR in the research laboratory, which contained racks filled with boxes of dates.

**Figure 3:**
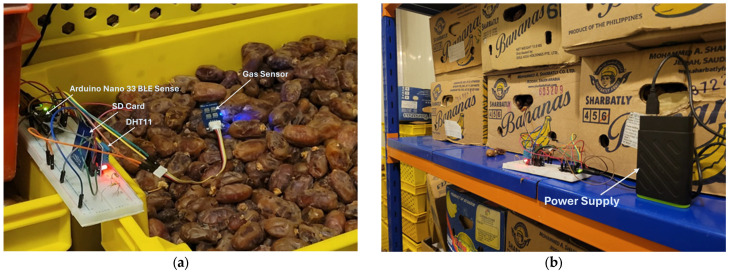
Measurement of gas concentration. (**a**) Room temperature collection. (**b**) Overall CSR data collection.

Figure 3 illustrates the experimental setup used for data collection under CSR and ambient conditions. Data were collected from the CSR, as shown in Figure 3a. Data were also collected in an ambient room temperature environment, as shown in Figure 3b, allowing us to conduct a comparative analysis. All these data were stored in SD card memory. The results of the analysis are compared with the actual data that are theoretically known or experimentally approved in agricultural research centers.

**Figure 4:**
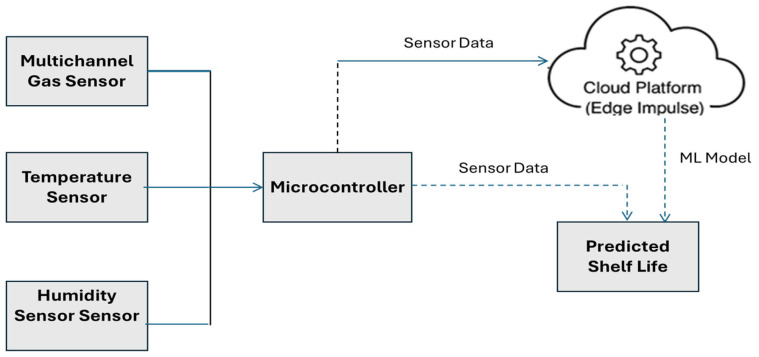
Dataflow architecture of the IoT-based shelf life estimation system.

In order to maintain signal stability and data consistency, the sensor system was configured to record readings at regular intervals of every 3 min. Data collection was conducted over an extended period in multiple phases, including initial testing followed by long-term monitoring. Prior to each reading, the system was allowed to stabilize within the storage environment to ensure that gas concentrations around the sensors had equilibrated. This procedure helped minimize transient fluctuations and ensured the acquisition of reliable and consistent data for model training and evaluation. The sensor system continuously collects environmental data from the CSR where date fruits are stored. This includes multichannel gas sensor readings for gases such as methane (CH_4_), carbon monoxide (CO), and nitrogen dioxide (NO_2_), along with temperature and relative humidity measured via dedicated temperature and humidity sensors. These readings are transmitted to a microcontroller (Arduino Nano 33 BLE Sense), which processes the incoming signals and prepares the data for local display, storage, or inference. The dataflow architecture is shown in Figure 4. During the offline training phase, raw sensor data is uploaded from the microcontroller to a cloud-based platform (Edge Impulse) [35], where a machine learning model is trained. Once trained, the optimized model is deployed back onto the microcontroller. In the online phase, the microcontroller performs real-time inference using the embedded model to predict the shelf life of stored dates based on current sensor input.

To enable shelf life prediction from gas sensor and environmental data, we employed Edge Impulse as the primary platform for data preprocessing, feature extraction, model training, and deployment. A sample of the collected gas concentration, temperature, and humidity readings is presented in Table 2. Although Table 2 presents 45 representative samples, the full dataset used for model training and evaluation was collected over several months. Sensor readings were taken every 3 min under both CSR and ambient room conditions, and features were extracted from aggregated time windows. The dataset was first uploaded to the Edge Impulse Studio, where data augmentation and filtering were applied to ensure consistency. Features were extracted using statistical methods, including mean, variance, and peak-to-peak values over sliding windows of sensor readings. These features were used to train a classification model using a fully connected neural network (FCNN) with ReLU activation functions. The model architecture consisted of an input layer matching the feature vector size, followed by two hidden layers (20 and 10 neurons, respectively), and a SoftMax output layer for three-class classification (Shelf Life High Type A, High Type B, and Low). The dataset was split into training (80%) and testing (20%) sets. Hyperparameter tuning was performed using validation accuracy and loss as optimization targets, and early stopping was used to avoid overfitting. The final model was validated using cross-validation and performance metrics such as precision, recall, F1-score, and AUC. For edge deployment, the trained model was quantized to 8-bit integer (int8) format using TensorFlow Lite, ensuring the real-time inference capabilities of the Arduino Nano 33 BLE Sense board.

The classification of shelf life categories, namely Shelf Life High Type A, High Type B, and Low, was based on a combination of the prior literature and expert input. Guidance was provided by experienced personnel at the Date Palm Research Center, who categorized the samples using their professional knowledge of postharvest quality traits, such as texture, odor, and appearance. This domain expertise helped to validate the labeling process used for training the machine learning model.

## 3. Results and Discussion

The dataset consists of gas sensor readings (GM102, GM302, GM502, GM702) and temperature and humidity values collected from stored date samples, providing critical insights into the fruit’s shelf life. The GM102 sensor values range from 404 to 429, potentially indicating variations in hydrogen (H_2_) emissions, which are closely linked to ripening and spoilage. GM302 readings, fluctuating between 176 and 198, likely reflect CO and NO_2_ concentrations, another key marker of fruit respiration and deterioration. Similarly, GM502 and GM702 values show fluctuations, capturing a broader spectrum of gas emissions that influence shelf life prediction. The temperature values range from 21.8 °C to 24.8 °C, reflecting natural ambient room temperature variations, while humidity remains stable at 33–34%, ensuring consistent monitoring conditions. The interplay between gas emissions, temperature, and humidity provides crucial data for training machine learning models aimed at predicting date shelf life with high accuracy, allowing for optimized storage conditions and reduced postharvest losses.

**Figure 5:**
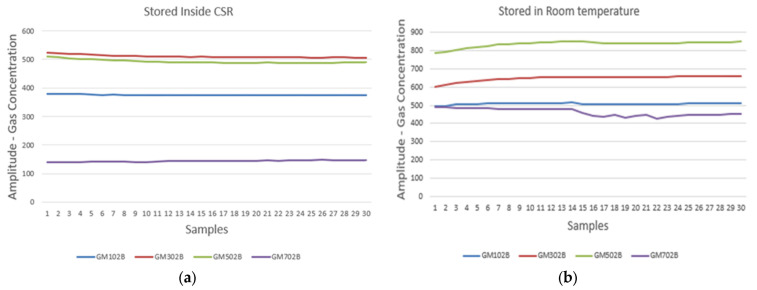
Gas concentration for 30 samples. (**a**) Measured in CSR environment. (**b**) Measured at room temperature.

Figure 5 provide an analysis of gas concentration stability under two distinct storage conditions, monitored using a multichannel gas sensor over 30 samples. Figure 5a illustrates the gas concentration levels for gases GM102B, GM302B, GM502B, and GM702B when stored within a CSR. The graph shows remarkably consistent gas concentrations with minimal variability, suggesting that the cold storage condition effectively stabilizes the gases, thereby mitigating any potential chemical degradation or interaction that could alter their concentrations. This stability is crucial for applications that require precise and reliable gas measurements, such as in determining the shelf life of dates.

In contrast, Figure 5b presents the same set of gases stored at room temperature. Here, the concentration levels exhibit more pronounced fluctuations, particularly for gases GM302B and GM502B. These variations could be attributed to environmental factors such as temperature changes, humidity, and exposure to ambient air, which can induce physical or chemical transformations in the gases. The graph indicates that, while GM102B and GM702B maintain relatively stable concentrations, GM302B and GM502B are more susceptible to changes, highlighting the need for the careful consideration of gas-specific storage requirements. These observations emphasize the critical role of storage conditions in maintaining gas quality and the functionality of gas sensors. By demonstrating the comparative stability of gases stored in CSR versus room temperature, the data advocates for tailored storage solutions based on the specific sensitivity and composition of each gas, ensuring accuracy and consistency in their use across various industrial and research settings.

**Figure 6:**
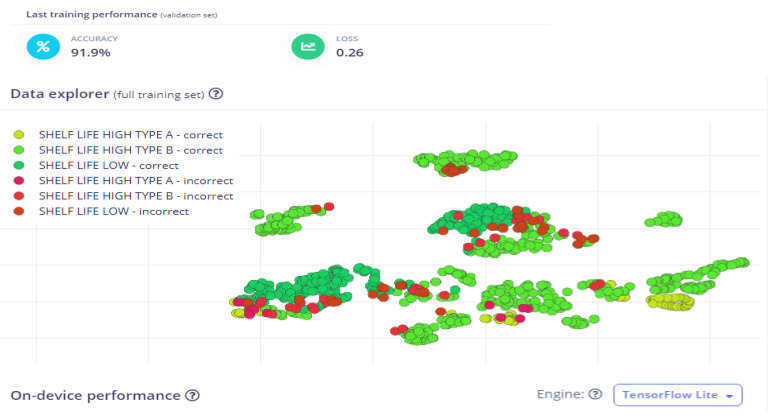
Data explorer visualization of the trained model’s classification results for shelf life estimation of dates.

Classification performance is shown in Table 3, where the model correctly classified 89.5% of Shelf Life High Type A samples. A visual summary of model prediction accuracy is presented in Figure 6 using a data explorer scatter plot. The trained machine learning model for the shelf life estimation of dates using a multichannel gas sensor demonstrated high classification performance, achieving a validation accuracy of 91.9% with a loss of 0.26, indicating a well-optimized model with minimal overfitting. The confusion matrix provides detailed insights into the model’s classification ability across three categories: Shelf Life High Type A, Shelf Life High Type B, and Shelf Life Low. The model correctly classified 89.5% of Shelf Life High Type A samples, with 5.3% misclassified as Type B and another 5.3% misclassified as Shelf Life Low. Similarly, Shelf Life High Type B exhibited the highest classification accuracy at 93.9%, with only 6.1% misclassified as Shelf Life Low. The Shelf Life Low category was accurately predicted in 87.7% of cases, but 12.3% of samples were incorrectly labeled as Shelf Life High Type B, suggesting minor overlaps in gas sensor readings between these classes. The weighted average precision, recall, and F1-score all stand at 0.92, highlighting the model’s ability to make reliable predictions while maintaining a strong balance between precision (minimizing false positives) and recall (minimizing false negatives).

The Receiver Operating Characteristic (ROC) curve yielded an AUC score of 0.98, confirming excellent model discrimination between shelf life categories, as per Table 4. This high AUC indicates that the model is highly effective in differentiating between fresh and deteriorating dates based on the multichannel gas sensor data. The data explorer visualization further validates the classification performance, where green points signify correctly classified samples, while red and yellow points represent misclassifications. A closer examination of the scatter plot reveals clusters of misclassified samples, particularly in transitional stages where the sensor readings of Shelf Life High Type B and Shelf Life Low overlap. This suggests that certain biochemical changes in dates, such as ethylene and CO_2_ emissions, may exhibit similar patterns between early and late spoilage stages, leading to minor misclassification errors.

From a deployment perspective, the model has been quantized to 8-bit integer format (int8) using TensorFlow Lite, significantly reducing its computational load while maintaining high accuracy. This optimization ensures efficient real-time performance on edge devices, making the model highly suitable for IoT-based cold storage monitoring systems. By integrating this machine learning model into automated food preservation workflows, it enables real-time decision-making to adjust storage conditions, reduce postharvest losses, and improve supply chain efficiency. Despite its high performance, further refinements—such as feature engineering, sensor recalibration, or ensemble modeling—could further reduce classification errors in edge cases where gas emission variations between shelf life stages are minimal. Nevertheless, with its high precision, recall, and real-time inference capabilities, this model provides a robust and scalable solution for intelligent date preservation and shelf life prediction.

## 4. Conclusions

This research presents a practical and cost-effective IoT-based framework that integrates multichannel gas sensing with TinyML for the real-time shelf life estimation of date fruits. By leveraging lightweight machine learning models deployed on edge devices, the system enables the accurate classification of date freshness based on spoilage-indicating gases and environmental parameters. An important contribution of this work is its non-invasive and scalable approach to freshness detection, eliminating the need for destructive testing or specialized packaging. The trained model achieved a classification accuracy of 91.9% and an AUC of 0.98, demonstrating strong predictive performance under both cold storage and ambient conditions. Designed with affordability and accessibility in mind, the architecture supports deployment on compact hardware such as the Arduino Nano 33 BLE Sense, making it highly applicable within real-world supply chains. Moreover, the system’s real-time monitoring and control capabilities facilitate their integration into both storage and transportation contexts, where early spoilage detection is critical to minimizing postharvest losses and enhancing food security. Future enhancements may include scaling the dataset, evaluating alternative machine learning models, and extending the methodology to other perishable commodities with minimal adaptation.

## Figures and Tables

**Table 1:** Modules and their functionalities.

Module	Model	Manufacturer	Function	Key Specs/Notes
Microcontroller	Arduino Nano 33 BLE Sense	Arduino	Central control, data processing	BLE, multiple onboard sensors, TinyML-ready
Gas Sensor	Multichannel Gas Sensor V2	Seeed Studio	Detects CH_4_, NO_2_, CO, NH_3_, H_2_, H_2_S	GM102, GM302, GM502, GM702 elements
Humidity and Temp Sensor	DHT11	Aosong Electronics	Measures temperature and relative humidity	Digital output, low cost
Real-Time Clock Module	DS3231	Adafruit	Timestamps data logs	I2C, battery-backed
SD Card Module	MicroSD Card Adapter	Generic	Local data storage	SPI Interface
OLED Display	SSD1306 0.96″	Adafruit/Generic	Displays real-time shelf life estimates	I2C, 128 × 64 pixels
Cloud Platform	Edge Impulse	Edge Impulse Inc.	ML model training and deployment	Edge ML support

**Table 2:** Sample dataset of gas sensor readings, temperature, and humidity.

Samples	GM102	GM302	GM502	GM702	Temperature	Humidity
1	429	198	153	228	22.6	33
2	424	194	150	227	22.6	33
3	421	191	147	227	22.2	33
4	418	188	145	226	22.2	33
5	420	188	145	227	22.2	33
6	422	189	147	228	23	33
7	424	192	149	230	23.4	33
8	426	193	151	230	23.8	33
9	428	194	152	229	24.1	33
10	428	196	154	230	24.5	33
11	423	194	152	227	23.8	33
12	419	191	148	225	23	33
13	417	188	146	225	22.2	33
14	413	186	143	223	22.2	33
15	413	184	142	223	21.8	33
16	416	184	143	224	22.6	33
17	417	186	144	226	23	33
18	419	188	147	227	23.4	33
19	420	189	148	227	23.8	33
20	421	190	150	227	24.1	33
21	421	190	150	226	24.1	33
22	415	187	147	222	23	33
23	413	185	145	222	22.6	33
24	410	183	141	221	22.2	33
25	408	181	139	219	21.8	33
26	409	180	139	220	21.8	34
27	411	181	140	222	22.6	33
28	413	183	142	223	23	33
29	414	184	143	222	23.4	33
30	416	186	145	224	23.8	33
31	417	187	146	225	24.1	33
32	415	187	147	222	23.8	33
33	411	184	144	220	23	33
34	407	182	141	219	22.6	33
35	405	180	138	219	22.2	33
36	402	177	137	219	21.8	34
37	405	178	136	219	22.2	34
38	408	179	138	220	22.6	33
39	410	181	140	221	23	33
40	411	182	141	221	23.4	33
41	412	183	143	222	23.8	33
42	414	184	144	223	23.8	33
43	411	184	144	221	23.8	33
44	407	181	142	218	23	33
45	404	179	139	217	22.2	33

**Table 3:** Confusion matrix analysis of the trained machine learning model.

Actual vs. Predicted	Shelf Life High Type A	Shelf Life High Type B	Shelf Life Low
Shelf Life High Type A	89.5% (correct)	5.3% (misclassified)	5.3% (misclassified)
Shelf Life High Type B	0% (misclassified)	93.9% (correct)	6.1% (misclassified)
Shelf Life Low	0% (misclassified)	12.3% (misclassified)	87.7% (correct)

**Table 4:** Metrics (validation set) for the trained machine learning model used in shelf life estimation.

Metric	Value
Area Under ROC Curve (AUC)	0.98
Weighted Average Precision	0.92
Weighted Average Recall	0.92
Weighted Average F1 Score	0.92

## Data Availability

The dataset is available upon request from the corresponding author.
